# Ykt6 mediates autophagosome-vacuole fusion

**DOI:** 10.1080/23723556.2018.1526006

**Published:** 2018-10-04

**Authors:** Levent Bas, Daniel Papinski, Claudine Kraft

**Affiliations:** a Max F. Perutz Laboratories, Vienna Biocenter, University of Vienna, Vienna, Austria; b Institute of Biochemistry and Molecular Biology, ZBMZ, Faculty of Medicine, University of Freiburg, Freiburg, Germany

**Keywords:** Autophagy, SNARE, fusion, Ykt6

## Abstract

Studying the mechanism of autophagosome-vacuole fusion has proven difficult in live yeast cells. Developing a novel *in vitro* fusion assay, we identified Ykt6 as the missing R-SNARE (Soluble *N*-ethylmaleimide sensitive factor attachment protein receptor) in this process and pinpoint the place of action of all four SNAREs involved. Parallel studies have confirmed our findings in other organisms.

Macroautophagy, hereafter referred to as autophagy, is a process for self-digestion of cytoplasmic components conserved in eukaryotes, which ensures cellular homeostasis. During autophagy, a double membrane engulfs autophagic cargo to form an autophagosome, which later fuses with the lytic compartment, i.e., vacuoles in yeast and lysosomes in higher eukaryotes, where the cargo gets degraded. The fusion step has been reported to depend on SNARE (Soluble *N*-ethylmaleimide sensitive factor attachment protein receptor) proteins. Its precise mechanism, however, had remained unknown. Generally, SNARE mediated fusion is achieved by the formation of a bundle of one R-SNARE and three Q-SNARE proteins, and the action of Rab GTPases and the HOPS (homotypic fusion and protein sorting) tethering complex. In yeast the Q-SNAREs Vam3, Vti1 and Vam7 had previously been shown to act during fusion, but whether these Q-SNAREs act on the autophagosome or the vacuole had remained unclear, as had the identity of the required R-SNARE.^1^ In order to identify this R-SNARE and the place of action of the known Q-SNAREs, and to dissect the molecular mechanism of the fusion process, we developed a novel autophagosome-vacuole fusion assay in which crude autophagosomes, vacuoles and a cytoplasmic extract complemented with ATP drive autophagosome-vacuole fusion *in vitro*.^2^

We found that in the cytosolic fraction, out of nine autophagy-related proteins (Atg) from six distinct parts of the autophagic machinery we tested, only Atg14 was required for autophagosome-vacuole fusion and that the activity of the Atg14-containing Phosphatidylinositol (PI) 3-kinase complex I is needed to recruit the Rab GTPase Ypt7 to the autophagosomal membrane. These findings were corroborated by Gao et al., who also detected Ypt7 on autophagosomes in a similar *in vitro* fusion assay.^3^ Also in *Drosophila melanogaster*, the Ypt7 homolog RAB7 is present on autophagosomes, depending at least in part on PI 3-phosphate.^4^

We found in our *in vitro* assay that, similar to other fusion processes, autophagosome-vacuole fusion depends on the HOPS tethering complex, as mutation of Vps11 – a subunit of the HOPS complex – abolished fusion. In line with our findings, Gao et al. observed an increase in the fusion efficiency when their *in vitro* system was supplemented with recombinant HOPS complex.^3^ Taken together, these findings suggest that the Rab GTPase Ypt7 on autophagosomal membranes and HOPS complex activity are required for autophagosome-vacuole fusion.

Next, we aimed at identifying where the Q-SNAREs Vam3, Vam7 and Vti1 act during the fusion step. It had been proposed that all three act on the vacuolar rather than the autophagosomal side, but formal proof for this model had been missing. Using our reconstitution assay, we found the presence of all three Q-SNAREs to be required in the vacuolar fraction, but neither on crude autophagosomes nor in the cytosolic fraction, providing evidence that they indeed act on the vacuole during autophagosome-vacuole fusion.

In yeast, the R-SNARE Ykt6 has been speculated to act during autophagosome-vacuole fusion. However, because Ykt6 mutants impair autophagosome formation, its role in autophagosome-vacuole fusion could not be studied in intact cells. Using our *in vitro* fusion assay, we were able to overcome this problem by using a temperature sensitive mutant of Ykt6. This allowed us to form the autophagosomes and prepare the autophagosome-enriched fraction at permissive temperature, but to block Ykt6 function by a shift to the restrictive temperature during the fusion step. We could show that Ykt6 is the missing R-SNARE for autophagosome-vacuole fusion, and that it acts on the autophagosomal membrane (Figure 1). In agreement with our findings, Gao et al. found Ykt6 to co-localize with the autophagosomal marker Atg8 *in vivo*, to be present on isolated autophagosomes, and fusion *in vitro* to be blocked in the presence of antibodies against Ykt6.^3^

**Figure 1.**
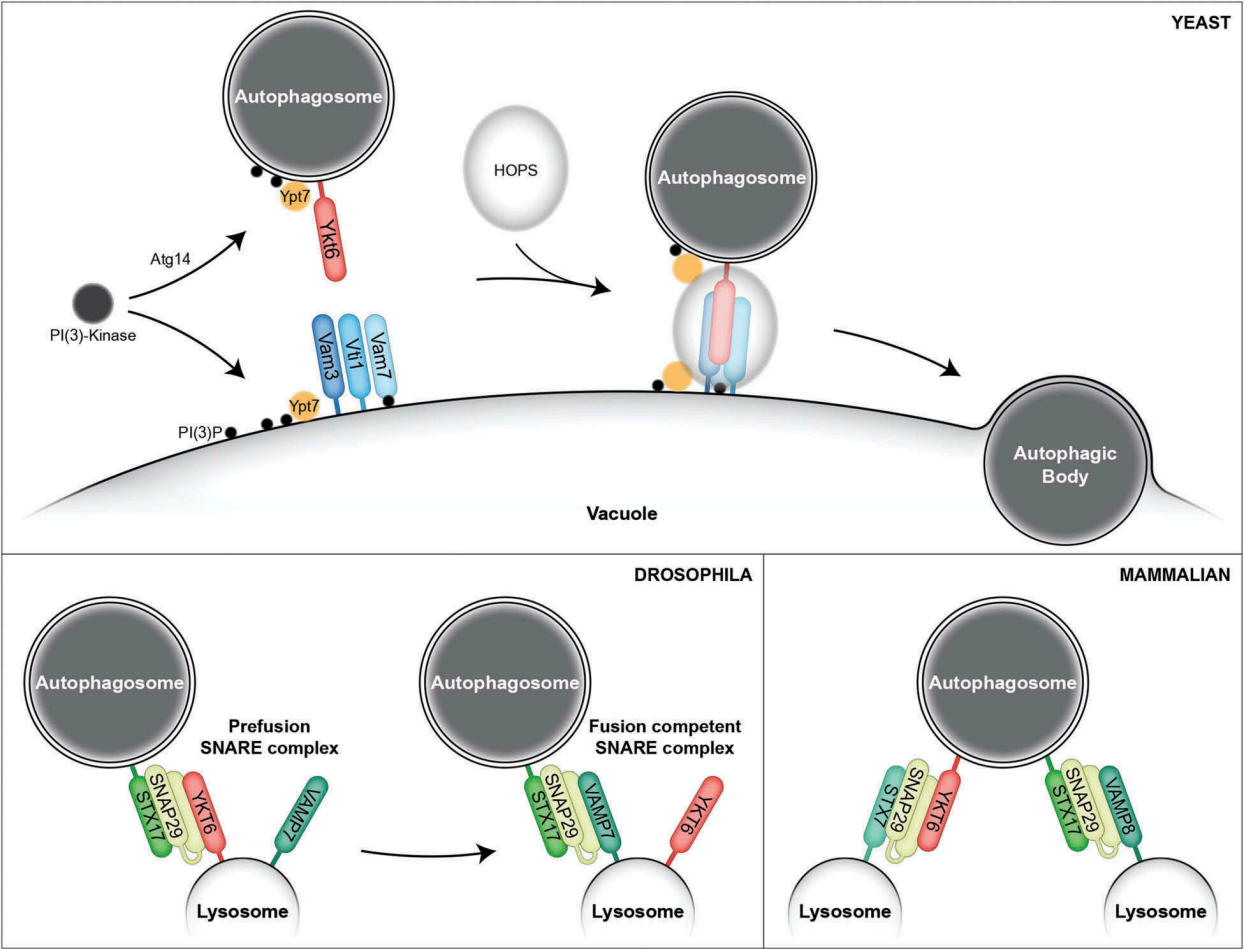
SNARE proteins in the fusion of autophagosomes with lytic compartments. In yeast, activity of the Atg14-containing Phosphatidylinositol (PI) 3-kinase complex I is required for the efficient recruitment of Ypt7 onto autophagosomal membranes. Vam3, Vti1 and Vam7 on the vacuolar membrane create a SNARE (Soluble *N*-ethylmaleimide sensitive factor attachment protein receptor) bundle with Ykt6 on the autophagosomal membrane, which is aided by Ypt7 on both membranes and the HOPS (homotypic fusion and protein sorting) tethering complex to allow fusion. In Drosophila, Ykt6 creates a fusion incompetent prefusion SNARE bundle with Stx17 and Snap29, which then is replaced by Vamp7 to form the fusion competent SNARE bundle. In mammalian cells, YKT6 and STX17 localize on autophagosomal membranes and create two separate SNARE bundles for autophagosome-lysosome fusion, with STX7 and SNAP29, and VAMP8 and SNAP29, respectively.

In higher eukaryotes, a bundle of different SNAREs has been reported to act during autophagosome-lysosome fusion: STX17, SNAP29 and VAMP7/8. Of these, only STX17 localises to the autophagosomal membrane.^5,6^ STX17 knock-out, however, does not abolish the fusion completely, and two independent screens for SNARE proteins in *D. melanogaster* and in mammalian cells showed that YKT6 is also required for autophagosome-lysosome fusion. In mammalian cells, STX17 and YKT6 act partially redundant in the fusion process: STX17 forms a SNARE bundle with SNAP29 and VAMP8, while YKT6 bundles with SNAP29 and STX7^7^ (Figure 1). STX17 and YKT6 were found to also interact with each other, but it is not yet known whether this interaction is of biological significance. In Drosophila, Ykt6 has been suggested to play a non-canonical regulatory role, acting before the bundling of Stx17, Snap29 and Vamp7, which promote fusion.^8^ Independently of its SNARE activity, Drosophila Ykt6 forms a prefusion complex with Stx17 and Snap29, before being replaced by Vamp7 to allow fusion (Figure 1). Whether YKT6 plays a similar regulatory role in mammalian cells remains to be investigated.

In summary, our work provides mechanistic information on the fusion step in autophagy. We show that the Q-SNAREs in yeast, Vam3, Vti1 and Vam7 act on the vacuolar membrane while the R-SNARE Ykt6 acts on the autophagosomal membrane. Together with the action of the Rab GTPase Ypt7 and the HOPS tethering complex, the Vam3-Vti1-Vam7-Ykt6 SNARE bundle facilitates the efficient fusion of autophagosomes with vacuoles. Having this *in vitro* assay in hands, we will not only be able to identify the components required in the the fusion process, but also to pinpoint where and how they act during this process.
